# The effect of hormone replacement therapy on cervical cancer risk in perimenopausal women: a systematic review and meta-analysis of observational studies

**DOI:** 10.3389/fonc.2025.1621570

**Published:** 2025-07-08

**Authors:** Yingna Zhou, Jie Wei, Youqin Ruan

**Affiliations:** The Department of Gynecology, Yunnan Cancer Hospital, The Third Affiliated Hospital of Kunming Medical University, Peking University Cancer Hospital Yunnan, Kunming, China

**Keywords:** perimenopausal women, hormone replacement therapy, cervical cancer, systematic review and meta-analysis, risk

## Abstract

**Background:**

Hormone replacement therapy (HRT) alleviates menopausal symptoms in perimenopausal women and help improve their quality of life, but its increased risk of cervical cancer (CC) remains to be evaluated.

**Methods:**

A system review and meta-analysis was conducted to retrieve literature related to HRT and CC risk by searching Pubmed, Embase, Science Direct, Web of Science, and Google Scholar databases. After screening the literature according to inclusion criteria and assessing the risk of bias using the Newcastle Ottawa Scale, the odd ratio (OR) values of HRT relative to CC were pooled.

**Results:**

A total of 9 articles were included in this study, including 5 cohort studies and 4 case control studies. The sample size of perimenopausal women in the literature ranged from 60 to 584,742. The overall quality of the literature was good. The meta-analysis results showed that HRT (current and persist use) had a reduced risk for CC (OR=0.70, 95% confidence interval (CI) [0.58, 0.85]), an increased risk for any cytological abnormality related to CC (OR=1.38, 95% CI [1.22, 1.55]), also an increased risk for adenocarcinoma of CC (OR=1.82, 95% CI [0.91,3.65]), but a decreased risk for squamous cell carcinoma of CC (OR=0.74, 95% CI [0.57, 0.96]). The subtype was a significant source of heterogeneity in this meta-analysis.

**Conclusion:**

HRT does not increase the overall risk of cervical cancer, but it increases the risk of cervical adenocarcinoma subtype and is associated with the risk of cancer-related cytological lesions.

## Introduction

Hormone replacement therapy (HRT) is a clinical intervention method adopted to address health issues caused by ovarian dysfunction and reduced sex hormone secretion due to various causes (1). Typically, the administration of HRT includes both estrogen (E) and progesterone (P). It has been revealed to significantly alleviate menopausal symptoms in postmenopausal women, including hot flashes, vaginal dryness and mood disturbance as depression. Evidence (2) also shows that it reduces the incidence rate of cardiovascular diseases and dementia. So far, HRT theramethodsains the most effective and common treatment method in managing menopausal symptoms, helping patients improve their quality of life, and facilitating a smooth transition to menopause (3).

However, the safety of HRT remains to be an ongoing topic. Patients administrated with HRT are susceptible to adverse symptoms such as nausea, appetite loss, hair loss, and an increased risk of gynecological diseases (4). A population-based large-scale of investigation (5) included 52,705 postmenopausal women with breast cancer and 108,411 without the disease reported that receiving HRT treatment for more than 5 years was associated with a 30% increased risk of breast cancer. A meta-analysis by Grady D et al. (6) also revealed that the relative risk of estrogen-use for endometrial cancer developing was much higher compared to non-users, and this relative risk increased with prolonged use.

Cervical cancer (CC) is one of the common gynecological malignancies among women. According to the cancer incidence statistics of the American Cancer Society (ACS) in 2023, CC has reached approximately 604,000 new cases worldwide per year, making it the post prevalent cancer of female reproductive system (7). The disease remains the leading cause of mortality among women in low- and middle-income countries (8). The development of this disease relates to multiple factors, including human papillomavirus (HPV) infection, immune suppression, and other contributing factors. The use of exogenous hormones has been suggested to induce malignant transformation of cervical epithelial cells, leading to the occurrence of this disease (9).

However, whether using HRT increases the risk of cervical cancer remains controversial. Reports on this topic show substantial variability. A case-control study by Parazzini F et al. (10) which included 40 CC patients and 86 controls suggested that the use of estrogen was associated with a reduced risk of CC by odd ratio (OR=0.5), whereas a cohort study by Anderson GL et al. (11) which included 16,608 postmenopausal women, reported current HRT users had a much higher incidence of cervical epithelial cell lesions compared with non-users (OR=1.44). Given meta-analysis represents an effective method to resolve the conflicting results, this study employed a meta-analytic approach to evaluate whether HRT use in postmenopausal women increases the risk of CC, aiming to provide evidence-based guidance for clinical decisions.

## Materials and methods

### Databases and search strategy

In April 2025, we searched Pubmed, Embase, Science Direct, and Web of Science databases using keyword free search mode, including keywords such as “Cervical neoplasms” or “Cervical cancer” or “Hormone replacement therapy” or “HRT”. We also searched for related topics on Google Scholar.

### Literature inclusion

Research type: all studies were observational studies including cohort studies which can be either retrospective or prospective, or case-control studies, or cross-sectional observational studies.

Research subjects: the research subjects were women who undergo HRT for premenopausal or postmenopausal women (including natural menopause, surgical or pharmacological menopause).

Object: the correlation between exposure (HRT) and outcome (risk of CC), should be studies in literature.

Outcomes: studies should provide the OR of HRT treatment relative to the risk of CC.

### Literature screening

Duplicate records were identified by using Endnote 20 (Build 16480) initially. Two researchers independently screened titles and abstracts based on predefined inclusion criteria. Discrepancies were resolved through structured discussion following objective criteria (1): re-examination against specific inclusion/exclusion criteria (2); consultation of the study protocol for borderline cases (3); involvement of a third reviewer when consensus could not be reached. All decisions and rationales were documented with justifications. The full texts of the potential studies (usually a PDF text) were retrieved one by one either from the online databases or by directly contacting the authors. Studies without available full texts were excluded after systematic attempts to obtain them through multiple strategies, including direct contact with corresponding authors, institutional library services, and inter-library loan requests. This exclusion of 11 studies (16.4% of studies sought for retrieval) represents a potential source of selection bias that may affect the generalizability of our findings. Then, the full texts were reviewed for further eligibility. Studies without outcomes or data would be excluded.

### Quality assessment

The Newcastle Ottawa Scale (NOS) (12) was used to assess the quality of the included studies. The scale evaluated three domains of observational studies including the object selection, comparability, and outcome indicators, with a maximum score of 9 points. Studies with a total score of 5 or above were of acceptable quality, score of 5–7 were of moderate quality, and 8–9 of high quality.

### Outcomes

Main outcome: the combined OR of HRT (current and persist use) for cervical cancer incidence.

Secondary outcomes: the combined OR of hormone replacement therapy (current and persist use) for any cytogenic abnormality associated with cervical cancer; The combined OR of past ever use hormone replacement therapy for cervical cancer incidence; The OR of hormone replacement therapy (Duration of use>1 year) for the incidence of cervical cancer; The OR of hormone replacement therapy (age>50) for cervical cancer incidence.

### Data extraction

Two researchers independently extracted literature data, including research type, publication date, research location, patient age, main indicators, etc., and then conducted data verification. Discrepancies were resolved through structured discussion with third reviewer consultation when necessary. All extracted data were cross-verified and documented.

### Effect size and pooling

The effect of HRT on the incidence of cervical cancer was evaluated using pooled OR and corresponding 95% CI. OR values reported in the literature were first log-transformed, then synthesized using the “metagen” function of the “meta” package, and the results were displayed in a forest plot. Model selection was based on heterogeneity assessment: fixed-effects model (Mantel-Haenszel method) was used when I²≤30% and *p*≥0.1 for Cochrane’s Q-test, indicating minimal heterogeneity and supporting the assumption of a common true effect size across studies. Random-effects model (DerSimonian-Laird inverse variance method) was used when I²>30% or *p*<0.1, indicating substantial heterogeneity and allowing for variation in true effect sizes between studies. The choice of I²=30% as a threshold was based on established guidelines that consider I²≤30% as representing low heterogeneity that may not substantially impact the pooled estimate.

### Heterogeneity statistics and source investigation

I^2^ analysis and Cochrane Q-test were used to detect heterogeneity between literature, with I^2^>30% or P<0.1 indicating heterogeneity in the results. The interpretation of heterogeneity levels followed established guidelines: I²=0-30% representing low heterogeneity, 31-50% moderate heterogeneity, 51-75% substantial heterogeneity, and 76-100% considerable heterogeneity (13). These thresholds guided our model selection process to ensure appropriate statistical methodology. Subgroup analysis was used to investigate the sources of heterogeneity between literature. Cluster analysis of literature based on heterogeneity and effect size was conducted using GOSH function (14).

### Sensitivity

The ‘metareg’ function from the ‘meta’ package was called to perform regression analysis, to detect whether the effect size is dominated by factors such as age, publication year, literature quality, and sample size. We simultaneously called the ‘influence’ function of the ‘metafor’ package to conduct influence analysis on the pooled results.

### Publication bias

The trim-and-fill method was used to predict the number of documents required to make the funnel fully symmetrical. Egger’s test was used to detect publication bias, and the results were presented in a funnel plot.

### Statistical analysis

All statistical analyses were performed using R software (version 4.4.1). Meta-analyses were conducted using the “meta” package (version 6.5-0) for effect size pooling and forest plot generation. Sensitivity analyses were performed using the “metafor” package (version 4.4-0). The GOSH (Graphical Display of Study Heterogeneity) function was implemented to explore potential clustering patterns among studies. Statistical significance was set at *p*<0.05 for all analyses.

## Results

### The result of literature selection

**Figure 1:**
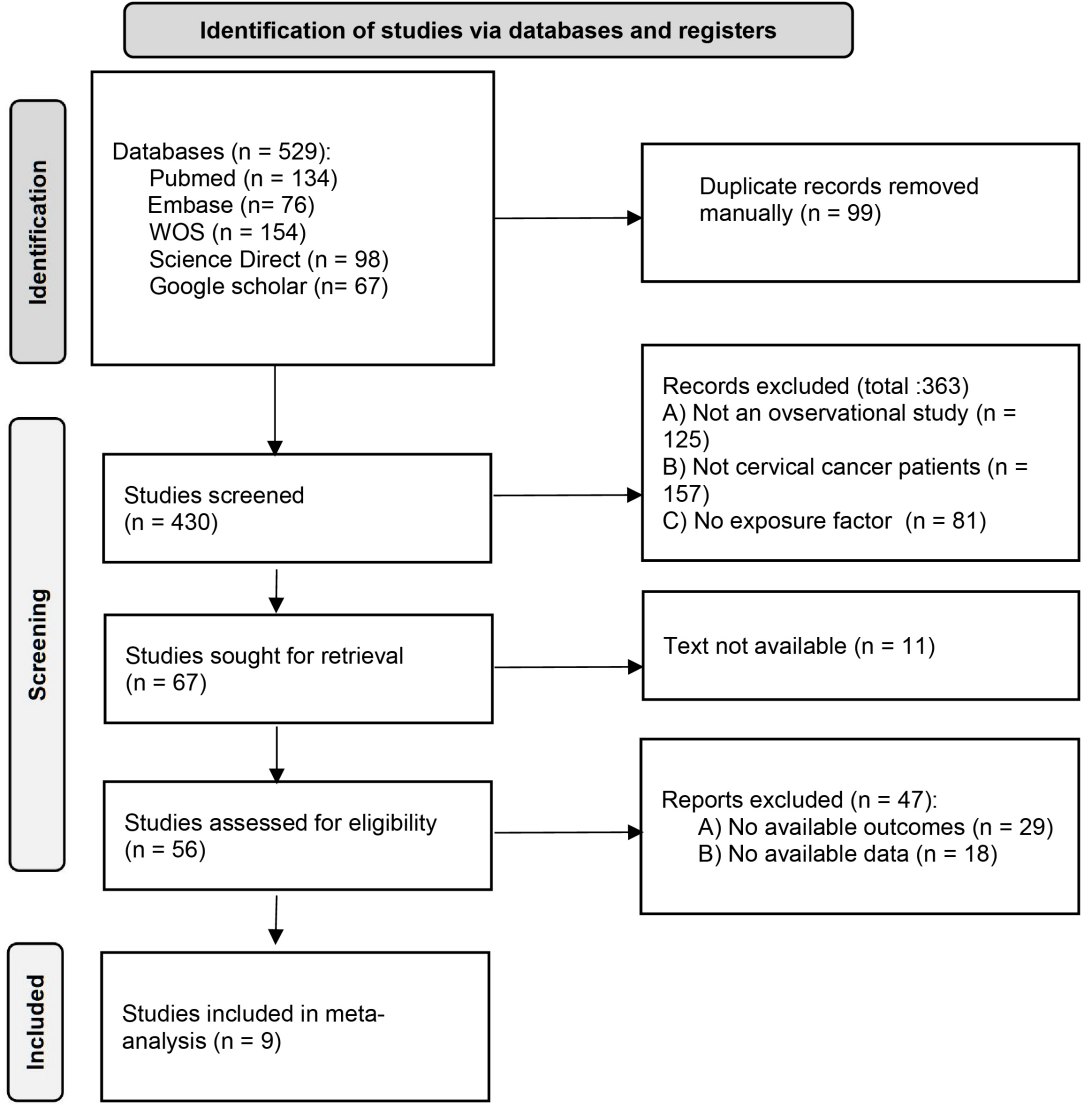
Study selection flow chart.

**Figure 1** shows the literature selection process. Initially, 529 articles were retrieved, and after preliminary duplication and screening, the PDF full texts of 56 articles were finally obtained. After detailed screening, 9 articles (10, 11, 14–20) were finally included.

### The characteristics of the included studies

The basic characteristics, grouping information, and outcome indicators of all literature and patients are listed in **Table 1**.

**Table 1:** Basic characteristics, participants, and outcomes of included studies.

Studies	Study type	Country	Samples	Case/controls	Age	Cancer type	Agents	Follow-up (years)	Indicators
Parazzini F et al. (10) 1997	Nested Case control	Italy	1,394	645/749	50-70	Any type	Estrogen alone	NR	OR
Anderson GL et al. (11) 2003	Prospective cohort study	US	16,608	8506/8102	50-79	Any type	Estrogen + progestin	5.2	HR
Lacey JV Jr et al. (15) 2000	Retrospective case control	US	570	263/307	NR	AC or SCC	Estrogen alone OR Estrogen + progestin	NR	OR
Adami HO et al. (16) 1989	Prospective cohort study	Sweden	653	–	54.5	Any type	Estrogens alone	4	RR
Roura E et al. (17) 2006	nested case-control study	EU	60	30/30	35-70	SCC	Estrogens alone OR Estrogen + progestin	9 (7,5, 10.8)	OR
Ahn KH et al. (18) 2009	Case control	Korea	4,996	268/4728	62.1 (31–90)	SCC	Estrogen + progestin	NR	OR
Yasmeen S et al. (19) 2006	Prospective cohort study	US	15,733	8070/7663	50-79	Any type	Estrogen + progestin	6	HR
Schneider C et al. (20) 2009	Prospective cohort study	UK	406	58/348	51.3 ± 6.1	Any type	Estrogen OR progestin	6	OR
Sakauchi F et al. (21) 2007	Prospective cohort study	Japan	584,742	10670/574072	NR	Any type	Estrogen + progestin	NR	HR

SCC, Squamous Cell Carcinomas. AC, Adenocarcinomas; E+P, Estrogen + progestin; E, Estrogens alone.

### Quality and bias assessment

Among all 9 articles included, 8 articles (89.9%) had a quality score of 8 or 9, with minimal bias and high quality; One article (1.11%) received a score of 7, with slight bias and moderate quality. The overall quality is good, as shown in **Table 2**.

**Table 2:** Bias risk assessment based on NOS of observational studies.

Studies	Patients selection(/4)	Comparability(/2)	Outcomes(/3)	Score(/9)
Parazzini F et al. (10) 1997	4	2	3	9
Anderson GL et al. (11) 2003	4	2	2	8
Lacey JV Jr et al. (15) 2000	4	2	2	8
Adami HO et al. (16) 1989	4	2	3	9
Roura E et al. (17) 2006	4	2	3	9
Ahn KH et al. (18) 2009	4	2	2	8
Yasmeen S et al. (19) 2006	4	2	2	8
Schneider C et al. (20) 2009	4	2	2	8
Sakauchi F et al. (21) 2007	3	2	2	7

NOS, Newcastle-Ottawa Scale.

### Effect size pooling

#### The combined effect of hormone replacement therapy (current and persist use) on cervical cancer incidence

**Figure 2:**
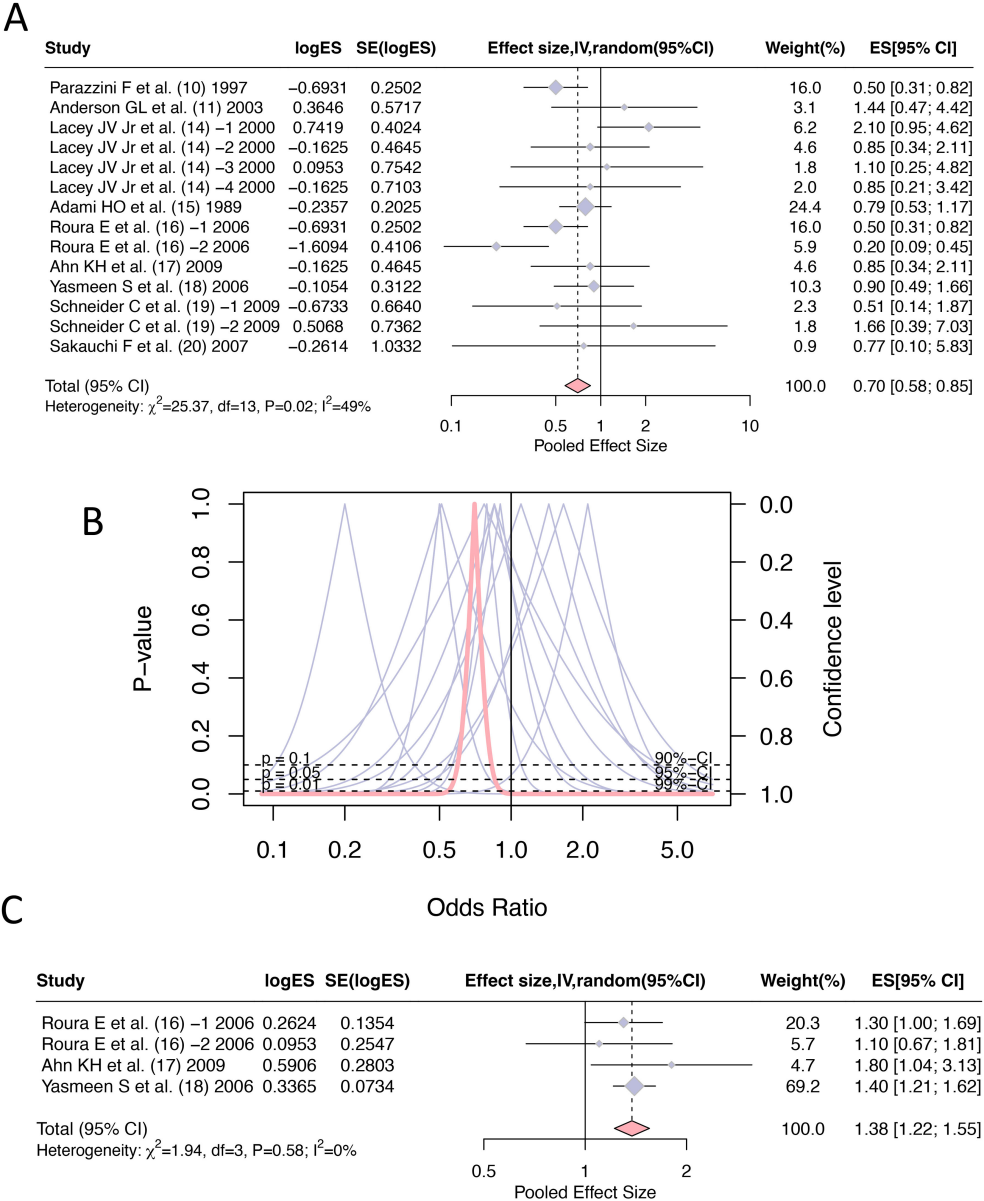
Forest plots of odds ratios for hormone replacement therapy (current and persistent use) and cervical cancer incidence: **(A)** Overall estimate; **(B)** Forest plot; **(C)** Any cytologic abnormality.

All literature reported the odd ratios of hormone replacement therapy (current and persist use) for the incidence of cervical cancer, with a total of 14 entries and a combined effect size: OR=0.70, 95% CI [0.58, 0.85]. The heterogeneity between the literature (I^2^ = 49%, *p*=0.02) was statistically significant, therefore, a random effect model was adopted during the merging process (**Figures 2A, B**). There was a total of 4 reports on the effect of hormone replacement therapy (current and persist use) on any cytologic abnormality related to cervical cancer, with a combined effect size: OR =1.38 and 95% CI [1.22, 1.55] (**Figure 2C**).

### Subgroup analysis and heterogeneity investigation

**Figure 3:**
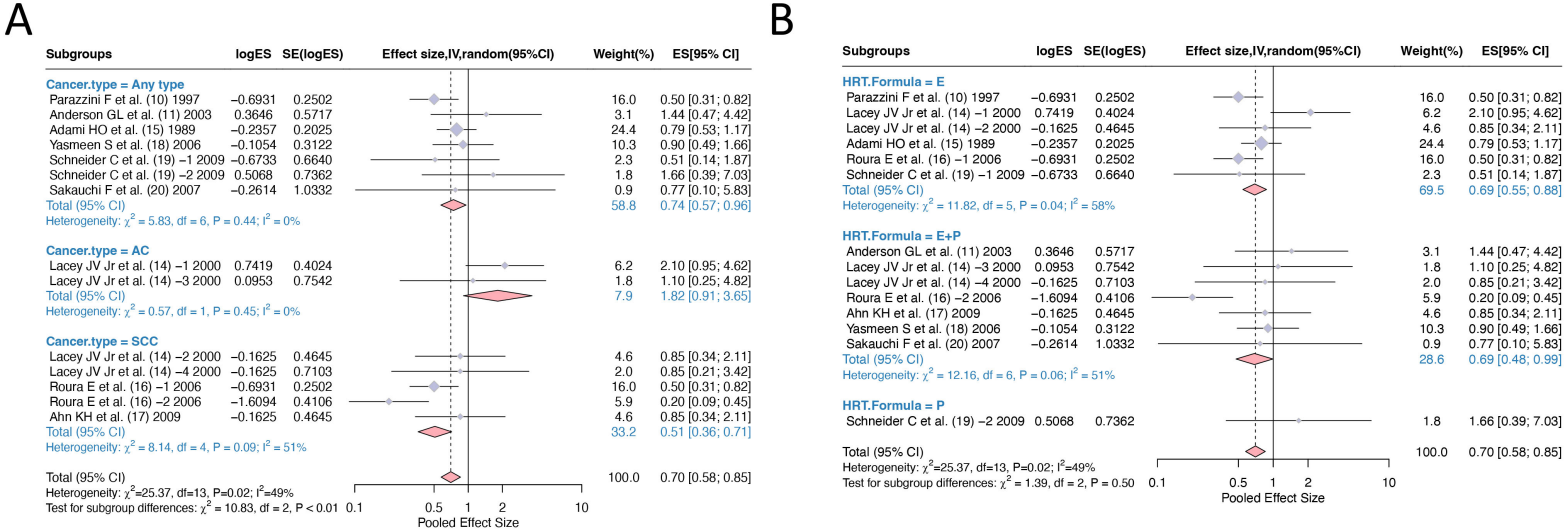
Subgroup analysis of odds ratios for hormone replacement therapy and cervical cancer incidence: **(A)** Grouping according to cancer type; **(B)** Grouping according to HRT formula. HRT, hormone replacement therapy.

In the pooling of ORs, there was statistical heterogeneity among 14 studies (I^2^ = 49%, *p*=0.02). We conducted subgroup analysis of the literature according to “Cancer type” and “HRT formula” to investigate the sources of heterogeneity. By dividing the studies into three subgroups according to ‘Cancer type’, the heterogeneity test between groups showed *p*<0.01, indicating ‘Cancer type’ is a statistically significant source of heterogeneity. The effect size of HRT for the ‘Any type’ cervical cancer subgroup was OR=0.74, 95% CI (0.57, 0.96), for adenocarcinoma subtype was OR=1.82, 95% CI (0.91, 3.65), while the effect size for squamous cell carcinoma subtype was OR=0.51, 95% CI (0.36, 0.71). Mixed type cervical cancer was mentioned in one study but had insufficient sample size for separate statistical analysis. These results suggest that HRT has a protective effect against cervical cancer overall and squamous cell carcinoma specifically but may increase the risk of adenocarcinoma subtype. According to the HRT formula, the studies can be divided into three subgroups with the following effect sizes: E alone OR=0.69, 95% CI (0.55, 0.88), E+P combined OR=0.69, 95% CI (0.48, 0.99), and P alone OR=1.66, 95% CI (0.39, 7.03). The heterogeneity test between subgroups showed *p*=0.50, indicating no significant heterogeneity between subgroups. These results demonstrate that both estrogen alone and estrogen plus progestin formulations have protective effects against cervical cancer, while progesterone alone shows a non-significant trend toward increased risk (**Figure 3**).

We also conducted subgroup analysis on the literature according to “Study design” and “Reported indicators” to investigate the sources of heterogeneity. According to the “Study design”, the two subgroups were identified, and the heterogeneity test between groups showed *p*=0.08, indicating that there was no significant heterogeneity between subgroups, ‘Study design’ is not a statistically significant source of heterogeneity. According to the “Reported Indicator”, the literature can be divided into three subgroups, and the heterogeneity test between subgroups was *p*=0.23, indicating the “Reported Indicator” is not a statistically significant source of heterogeneity (**Table 3**).

**Table 3:** Subgroup analysis evaluated by study design and indicators.

Index	Group method	Subgroups	Number	Effect size	Intra group heterogeneity		*p*-value of inter group heterogeneity test
					I^2^	P	
1	Study design	Cohort study	6	OR = 0.86, 95%CI [0.58; 0.85]	0%	0.79	0.08
		Case control study	8	OR = 0.60, 95%CI [0.47; 0.78]	62%	<0.01	
2	Reported indicator	OR	10	OR = 0.62, 95%CI [0.48; 0.82]	59%	<0.01	0.23
		HR	3	OR = 0.99, 95%CI [0.59; 1.66]	0%	0.75	
		RR	1	OR = 0.70, 95%CI [0.58; 0.85]	–	–	

### The combined effect of hormone replacement therapy with other characteristics on the incidence of cervical cancer

**Figure 4:**
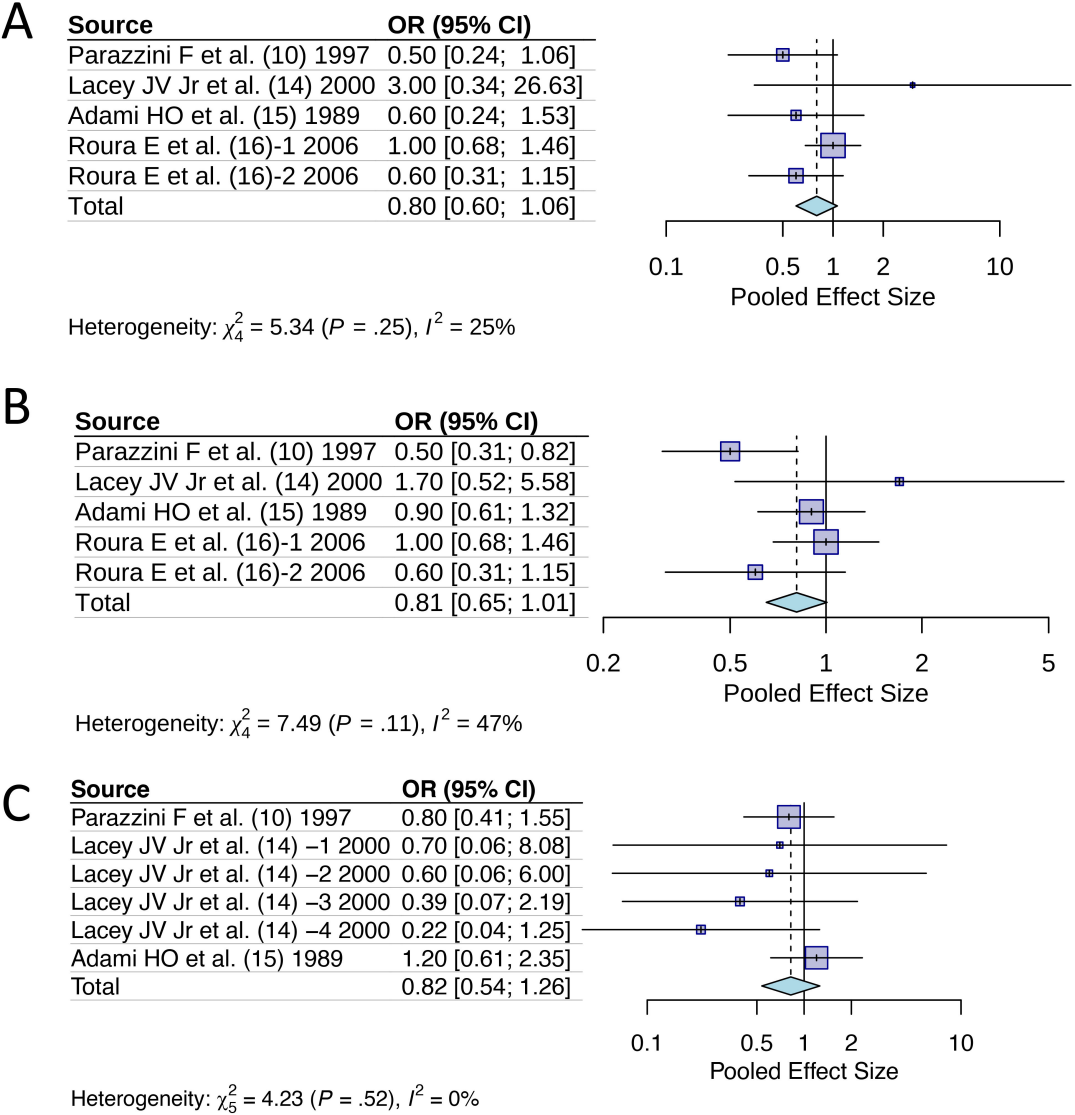
Odds ratios of hormone replacement therapy with specific features for cervical cancer incidence: **(A)** Past ever use; **(B)** Duration of use >1 year; **(C)** Age >50 years.

We conducted additional analyses to examine the effect of specific HRT characteristics on cervical cancer risk (**Figure 4**). For past use of HRT, analysis of 5 studies revealed a pooled OR of 0.80 (95% CI [0.60, 1.06]), suggesting a protective trend that did not reach statistical significance (*p*>0.05). This finding indicates that even previous HRT exposure may confer lasting protective effects against cervical cancer development. Duration-specific analysis of 5 studies examining HRT use >1 year showed an OR of 0.81 (95% CI [0.65, 1.01]), demonstrating consistent protective effects regardless of treatment duration, with the upper confidence limit approaching but not exceeding unity. Age-stratified analysis of 6 studies focusing on women >50 years yielded an OR of 0.82 (95% CI [0.54, 1.26]), indicating that HRT’s protective effect persists across different age groups, though with wider confidence intervals reflecting increased heterogeneity in older populations. Notably, all three analyses showed remarkably consistent effect sizes (OR = 0.80-0.82), reinforcing the robustness of HRT’s protective association against cervical cancer across different patient characteristics and exposure patterns.

### Meta-regression analysis

**Figure 5:**
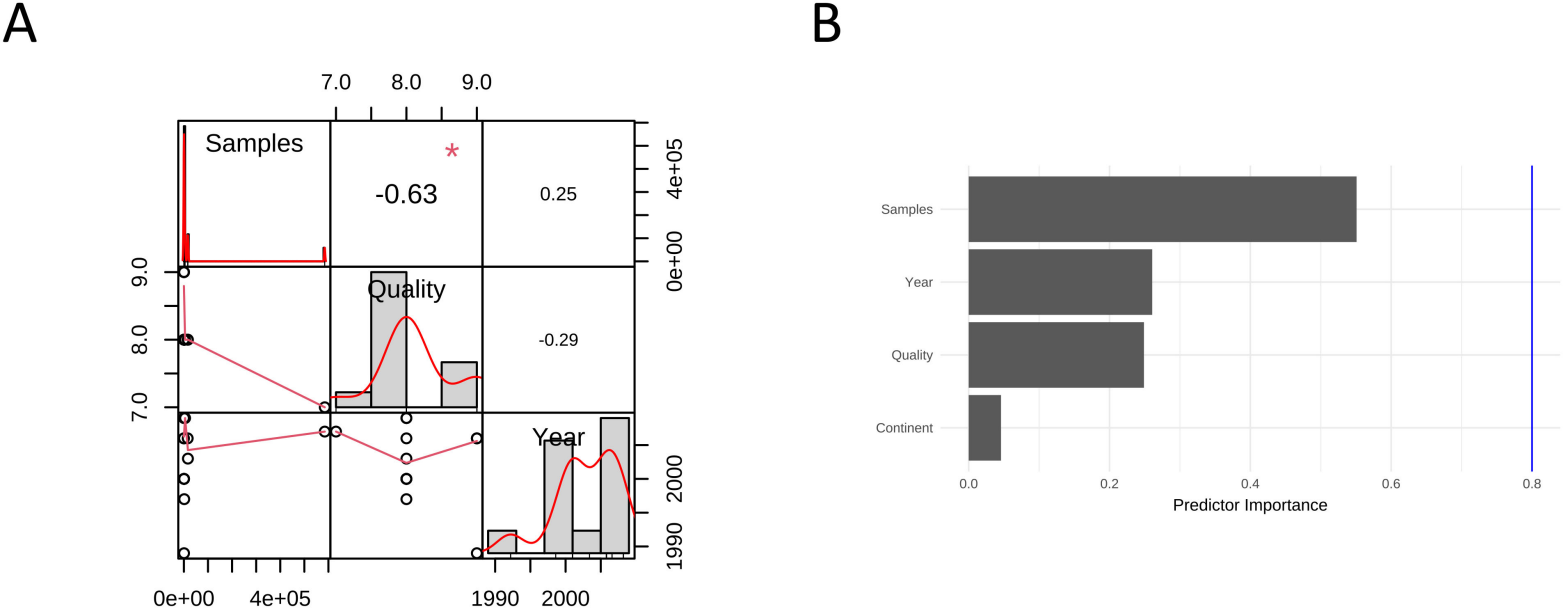
Meta-regression analysis of odds ratios for hormone replacement therapy and cervical cancer incidence: **(A)** Correlation between variables; **(B)** Importance of 4 predictors.

To investigate the factors that may affect the pooled ES results, we conducted correlation analysis using three sets of data: “publication year”, “sample size”, and “literature quality score” and found a strong correlation of -0.63 between sample size and literature quality (**Figure 5A**). We conducted a multivariate regression analysis using four conventional variables: ‘publication year’, ‘sample size’, ‘literature quality score’, and ‘Continent’ and found that none of these factors had a statistically significant impact on the results (*p*>0.05). Based on our subgroup analyses, cancer subtype appears to be the primary dominant variable, showing significant between-group heterogeneity (*p*<0.01). While HRT formulation did not show significant heterogeneity between subgroups (*p*=0.50), other potentially dominant variables that warrant future investigation include HRT duration, mean participant age, baseline HPV status, and menopausal status, as these clinical and biological factors may have stronger influence on cervical cancer risk than the conventional methodological variables we initially examined. Cancer subtype is currently the only confirmed dominant factor based on our available data, highlighting the need for more comprehensive variable collection in future meta-analyses (**Figure 5B**).

### Sensitivity analysis

**Figure 6:**
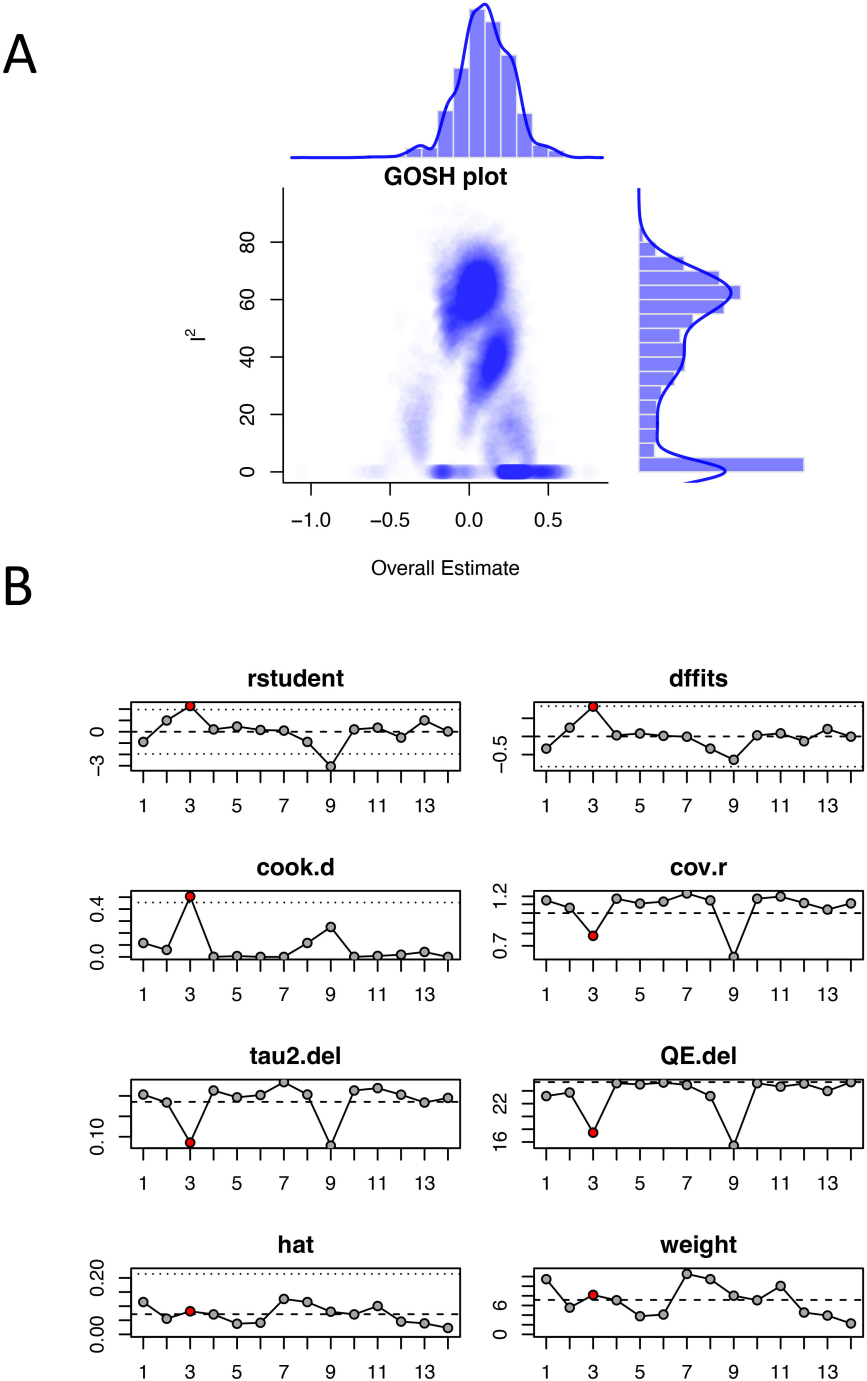
Sensitivity analysis of odds ratios for hormone replacement therapy and cervical cancer incidence: **(A)** GOSH plot; **(B)** Influence analysis plot. The red dots represent outlier studies.

The GOSH diagnosis clearly clustered the studies into 3 groups. Based on the results of previous subgroup analysis, we speculate that these clusters were related to different types of cervical cancer (**Figure 6A**). Influence analysis using multiple diagnostic statistics identified literature (15) [study (3) in **Figure 6B**] as an influential outlier across several measures including standardized residuals (rstudent), DFFITS values, Cook’s distance (cook.d), and covariance ratios (cov.r). The study exceeded conventional threshold criteria across these diagnostics, as indicated by the red triangular markers in **Figure 6B**. However, sensitivity analysis excluding this study showed minimal change in the pooled effect size, confirming the robustness and reliability of our meta-analysis results.

### Publication bias

**Figure 7:**
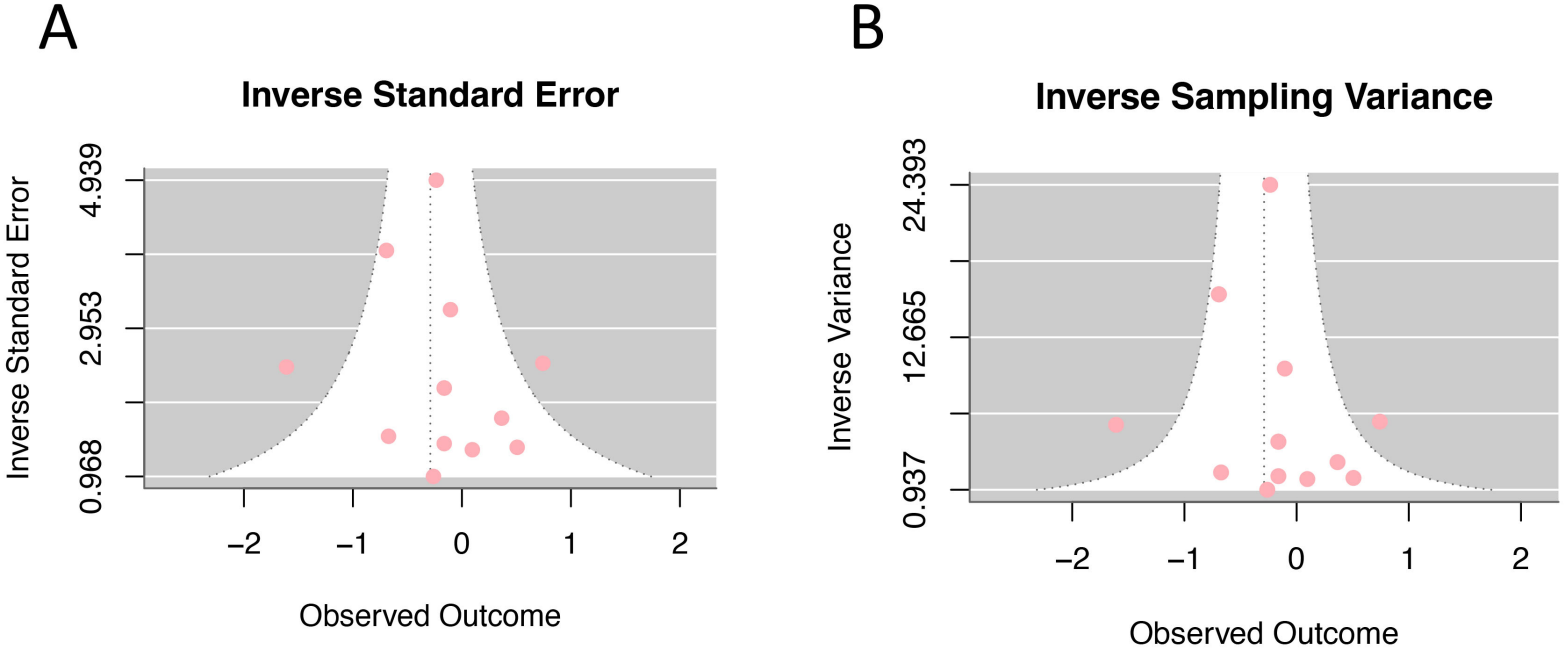
Funnel plot of odds ratios for hormone replacement therapy (current and persistent use) and cervical cancer incidence: **(A)** Inverse standard error; **(B)** Inverse sampling variance.

In the analysis of the effect size of HRT (current and persist use) for cervical cancer incidence, 14 studies were assessed using the trim-and-fill method. The result suggested an additional 5 studies need to be added to fully compensate for the asymmetry on both sides of the funnel. However, Egger’s test yielded *p*=0.386, indicating that the asymmetry on both sides of the funnel is not statistically significant. The unadjusted funnel plots of 14 studies were shown in **Figure 7**.

## Discussion

HRT is the most effective treatment for menopausal syndrome currently, offering significant relief from symptoms of menopause in women and improvement of their quality of life (22). However, the potential association between HRT and the risk of female malignancies has always been a big concern among researchers (23). This meta-analysis represents the first comprehensive quantitative synthesis examining HRT effects on cervical cancer risk with subtype-specific analysis. Our findings demonstrate an overall protective effect, which contrasts sharply with established HRT associations in other gynecological malignancies such as breast and endometrial cancers where increased risks are consistently reported (24, 25). This divergent pattern suggests distinct hormonal sensitivity mechanisms in cervical carcinogenesis.

The OR values of all the 9 studies were pooled and a combined estimate of OR=0.70 was obtained, indicating that overall, the use of external hormones is still safe for the occurrence of cervical cancer. In fact, an OR<1 suggests a decrease in the incidence of cervical cancer, and the use of exogenous hormones has a protective effect on cervical cancer. Additionally, we pooled the results of any reported cytological lesions, and yielded a combined estimate of OR=1.38, which suggested that although exogenous hormones did not increase the incidence rate of CC, they may be associated with a likelihood of developing cytological lesions in the cervical epithelium.

The observed protective effect likely involves multiple interconnected mechanisms. Estrogen’s immunomodulatory properties may enhance Langerhans cell function and improve local immunosurveillance against HPV infection (26). Additionally, HRT users typically undergo more frequent gynecological surveillance, enabling earlier detection and treatment of precancerous lesions (27). The differential subtype effects reflect distinct biological pathways: cervical adenocarcinoma demonstrates estrogen-receptor positivity like endometrial cancer, while squamous cell carcinoma benefits from enhanced immune surveillance, which is a high-risk factor for epithelial cell related cancer (28, 29). In addition, studies (30) have observed that patients who take exogenous hormones for a long time have a significant decrease in the number of Langerhans cells in the cervical squamous epithelial junction, leading to local tissue immunodeficiency and inability to prevent potential diseased cells from developing into cervical tumor cells. But from the long run, the incidence rate of CC has not increased. We speculate that the protective effect of HRT may be due to the stricter medical control measures in the population using HRT, the regular follow-up and screening for cervical exfoliated cells. Therefore, early detection and treatment of benign or precancerous cervical lesions can be achieved, which to some extent prevents the occurrence of cervical cancer (31).

Subgroup analysis by histological type revealed important differential effects. Mixed type cervical cancer was identified in the literature but had insufficient data for meaningful separate analysis. The results showed that the use of exogenous hormones had a significantly increase in the incidence rate of adenocarcinoma, but a decrease in squamous cell carcinoma, indicating differential hormonal sensitivity between cervical adenocarcinoma and squamous cell carcinoma (32). Currently, evidence generally supports the view that cervical squamous cell carcinoma is not hormone dependent and can be safely treated with HRT. In contrast, cervical adenocarcinoma appears to be hormonally sensitive, and the use of HRT may not only be associated with the development of the disease but also contribute to the prognosis of it (33). These findings are consistent with the results of this study. Therefore, patients with precancerous lesions or those who have been diagnosed with cervical adenocarcinoma should be particularly cautious when using HRT.

Our findings should be interpreted in the context of existing systematic reviews examining HRT and cervical cancer relationships. The recent systematic review by Vargiu V et al. (34) evaluated the safety of HRT in cervical cancer survivors and concluded that HRT is generally not contraindicated after cervical cancer treatment, demonstrating protective effects against cervical squamous cell carcinoma. Our quantitative meta-analysis strongly supports these conclusions, showing an overall protective effect (OR=0.70) and specific protection for squamous cell carcinoma (OR=0.51). Importantly, both studies consistently demonstrate that HRT does not increase overall cervical cancer risk. While Vargiu et al. conducted a qualitative systematic review, our study provides the first comprehensive quantitative synthesis with precise pooled effect estimates and subtype-specific risk assessments.

We also conducted subgroup analysis according to the composition of HRT. The results showed that the use of E alone or the combination of E+P did not increase the incidence rate of cervical cancer, but the use of progesterone P alone might be associated with the increased risk. However, it should be noted that this finding is based on a single study, which limits the level of evidence. The International Agency for Research on Cancer (IARC) has previously reported (35) that the use of E alone by postmenopausal women will increase the risk of gynecological malignancies including breast cancer and endometrial cancers. Therefore, the combined E+P regimen is recommended for HRT in perimenopausal women, from a safety perspective.

Our analysis of HRT characteristics provides important clinical insights into the consistency of protective effects across different patient populations and treatment patterns. The remarkable consistency of effect sizes across past ever use, duration >1 year, and age >50 years suggests that HRT’s protective association with cervical cancer is robust and independent of these patient characteristics (36). This consistency is particularly important for clinical decision-making, as it indicates that the protective effect is not limited to specific treatment durations or patient age groups. The finding that past ever use maintains protective trends suggests potential lasting benefits even after treatment discontinuation, which has important implications for long-term cancer risk assessment in former HRT users (37). The age-stratified analysis showing maintained protective effects in women >50 years is clinically significant, as this population represents the primary target group for HRT therapy.

To assess the sensitivity of the results, we conducted regression analysis on four potential influencing factors “publication year”, “sample size”, “geographic region (continent)”, and “literature quality”. We found that none of these four factors dominated the overall meta-analysis estimate. Additionally, GOSH diagnosis revealed that the studies could be clustered into about 3 groups, potentially reflecting differences in subtypes of cancer. In the influence analysis, reference (15) was identified as an outlier. However, the overall estimate was not significantly altered after excluding this reference, indicating that the findings of this meta-analysis are stable and robust.

The trim-and-fill method found that approximately 5 additional references should be needed to achieve full symmetry in the funnel plot. However, Egger’s test did not conclude significant asymmetry, indicating that the publication bias of this study is minimal. The evidence level is strengthened by the large sample sizes of the included studies and the high quality of them. Nevertheless, several limitations should be acknowledged. First, excluding 11 studies due to unavailable full texts may introduce selection bias, as these studies might systematically differ from included ones in outcomes, geographic distribution, or methodology, potentially affecting result generalizability. Second, the total number of studies included was still relatively small; second, all studies included were published between 1997 and 2009, and there has been a notable lack of studies reporting on this topic over the past decade. Therefore, further high-quality, rigorous designed clinical controlled studies are needed to provide more robust evidence. Important limitations include the lack of standardized long-term duration reporting (>5 years) and inconsistent HPV status documentation across studies. Future research should prioritize standardized long-term follow-up and systematic HPV status reporting to enable comprehensive risk stratification and better comparability with other hormonal contraceptive studies.

## Conclusion

The findings of this meta-analysis suggest that HRT does not increase the overall risk of cervical cancer, but it may elevate the risk of developing cervical cytological lesions, and the risk of cervical adenocarcinoma is significantly higher than other subtypes. In clinical practice, healthcare providers should educate perimenopausal women about the knowledge of HRT, promote standardized medication use of it, and emphasize the importance of regularly screening for cervical cells, and prevent potential adverse effects during HRT application.

## Data Availability

The original contributions presented in the study are included in the article/supplementary material. Further inquiries can be directed to the corresponding author.
